# Advances in Fungal Elicitor-Triggered Plant Immunity

**DOI:** 10.3390/ijms231912003

**Published:** 2022-10-09

**Authors:** Jia Guo, Yulin Cheng

**Affiliations:** Key Laboratory of Plant Hormones and Development Regulation of Chongqing, School of Life Sciences, Chongqing University, Chongqing 401331, China

**Keywords:** fungal elicitors, plant immune system, PTI, ETI, receptors

## Abstract

There is an array of pathogenic fungi in the natural environment of plants, which produce some molecules including pathogen-associated molecular patterns (PAMPs) and effectors during infection. These molecules, which can be recognized by plant specific receptors to activate plant immunity, including PTI (PAMP-triggered immunity) and ETI (effector-triggered immunity), are called elicitors. Undoubtedly, identification of novel fungal elicitors and their plant receptors and comprehensive understanding about fungal elicitor-triggered plant immunity will be of great significance to effectively control plant diseases. Great progress has occurred in fungal elicitor-triggered plant immunity, especially in the signaling pathways of PTI and ETI, in recent years. Here, recent advances in fungal elicitor-triggered plant immunity are summarized and their important contribution to the enlightenment of plant disease control is also discussed.

## 1. Introduction

Plant diseases are a major problem affecting the yield and quality of crops, and annual losses amounted to 10.1–28.1% for wheat, 24.6–40.9% for rice, 19.5–41.1% for maize and 8.1–21% for potatoes [1]. Pathogenic fungi are the most frequent cause of plant diseases and over 8000 fungal species are capable of infecting plants [2]. In addition, some pathogenic fungi contain toxins, such as aflatoxins and fumonisins, which are harmful to human health [2]. Pathogenic fungi are a persistent threat to global food security, and the application of fungicides is still the main method to control fungal diseases worldwide [3].

Investigating the molecular mechanisms of plant-microbe interactions, mainly including pathogenicity and plant resistance, is crucial for developing novel or safer strategies for effectively controlling plant diseases [4]. For infecting plants, pathogens can produce some important virulence factors, such as cell wall-degrading enzymes, virulence-associated effector proteins and phytotoxins, to promote infection [5]. To survive from biotic stresses, plants have also evolved an extraordinary immune system over the long term by interacting with pathogens [6]. Unlike animals, plants have innate immune systems that perceive danger signals and trigger defense responses, mainly including reactive oxygen species (ROS) burst, ion flow, accumulation of phytoalexins, upregulation of immune genes, hypersensitive response (HR), and systemic acquired resistance (SAR), which play a central role in plant resistance against pathogen infection [7,8].

Elicitors are a general term for a group of substances that act on plants and improve their resistance, and they can be classified into biotic elicitors and abiotic elicitors depending on their source [9]. During pathogen infection on plants, some molecules from pathogens are perceived by plants as danger signals to activate plant immunity. These molecules belong to biotic elicitors and have great application prospects in plant disease control [10]. Pathogen-derived elicitors mainly consist of pathogen-associated molecular patterns (PAMPs) and effectors, which can be recognized by plant cell surface-localized pattern recognition receptors (PRRs) and intracellular nucleotide-binding leucine-rich repeat (NB-LRR) domain receptors (NLRs) to activate PTI (PAMP-triggered immunity) and ETI (effector-triggered immunity), respectively [6,11]. PAMPs are also called microbe-associated molecular pattern (MAMPs), and a well-known fungal PAMP is chitin, from the fungal cell wall [12]. Effectors are secreted by pathogens to enter host plants, and the effectors activating ETI are also called avirulence (AVR) proteins. In recent years, a number of fungal elicitors and their plant receptors have been identified and great progress has occurred in the signaling pathways of fungal elicitor-triggered plant immunity. Here, these recent advances in fungal elicitor-triggered plant immunity are summarized. 

## 2. Classification of Fungal Elicitors

**Figure 1:**
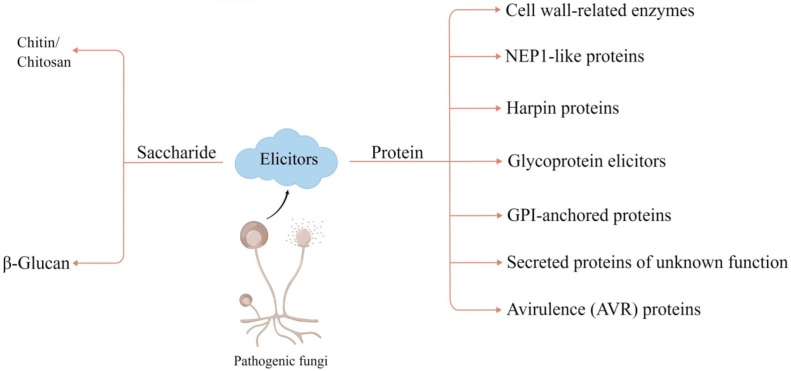
Classification of fungal elicitors. Known fungal elicitors can be classified into two major categories, including saccharide elicitors and protein elicitors. The figure is drawn by Figdraw.

According to their chemical composition, known fungal elicitors can be classified into two major categories, including saccharide elicitors and protein elicitors (Figure 1; Table 1 and Table 2). Chitin and cell wall-degrading enzymes are well-known fungal elicitors, and they belong to oligosaccharide elicitors and protein elicitors, respectively.

### 2.1. Saccharide Elicitors

Chitin is one of the key structural components in the fungal cell wall and chitosan is the production of deacetylated chitin [13]. Despite the low content (1~2% in dry yeast cell wall, 10~20% in the cell wall of filamentous fungi), chitin is studied as a model of fungal PAMPs, and it can be recognized by specific receptors, resulting in activating downstream immune signals [14]. As a derivative of chitin, chitosan can also induce immune response-like chitin [15]. Plants do not contain chitin, but contains chitin-degrading enzymes, which can degrade the cell wall of the fungi, and the resulting products, chitin oligosaccharides and chitosan oligosaccharides, can act as elicitors to activate the plant immune system, inducing ion fluxes, increase of chitinase activity, synthesis of phytoalexins, production of ROS and expression of defense genes [16]. Receptors containing the lysin motif (LysM) located on the surface of plant cells can recognize chitin in different plants [17,18]. 

Glucan is a type of polysaccharide, made up of glucose linked by glycosidic bonds, and glucan fragment obtained from the hydrolysis of glucan has elicitor activity [19]. The recognition to chitin is conserved in plants while the recognition to glucan is dependent on its origin and the species of plants [14]. Glucan, with two forms including α-glucan and β-glucan, is an important structural component and the most abundant polysaccharide in cell walls of fungi [20]. Among all the β-glucan of fungal cell wall, β-1,3-glucan has the highest percentage ranging from 65 to 90 percent, which binds to β-1,6-glucan in the form of a branch [21,22,23]. Ayers et al. found that β-glucan could enhance resistance in plants as a PAMP by promoting the accumulation of phytoalexins and improving their activity against pathogen infection [24]. Researchers showed that a large number of legumes including soybean, alfalfa, bean, lupin, and pea had a specific receptor in their membrane to bind with β-1,3/-1,6-glucan heptaglucoside [25,26]. Rebaque et al. have found that mixed-linked β-1,3/1,4-glucans (the smallest structure is MLG43) induce MLG43-triggered PTI immunity in Arabidopsis, partially dependent on some known plant PRRs, including CERK1, LYK4 and LYK5 [27]. During the interaction between plants and β-glucan, immune responses such as ROS production, MAPKs activation, and defensive gene expression can be observed [27].

### 2.2. Protein Elicitors

Compared to saccharide elicitors, protein elicitors appear to be relatively diverse and mainly include cell wall-related enzymes (CWDEs), NEP1-like proteins (NLPs), harpins, glycoprotein elicitors, GPI-anchored proteins, secreted proteins of unknown function, and AVR proteins (Figure 1; Table 1 and Table 2). 

#### 2.2.1. Cell Wall-Related Enzymes 

Pathogens secrete cell wall-related enzymes, most of which are cell wall-degrading enzymes (CWDEs), to degrade important components of plant cell walls in order to draw nutrients from plants or maintain the integrity of fungal pathogens [28,29]. Fungal cell wall-related enzymes may be recognized by plants as elicitors, enhancing plant resistance to pathogenic fungi, and they mainly include the glycoside hydrolase (GH), carbohydrate esterase (CE) and polysaccharide lyase (PL) families. GHs-elicitors were identified in many plant pathogenic fungi, such as *Botrytis cinerea*, *Colletotrichum lindemuthianum*, *Fusarium graminearum*, *Rhizoctonia solani*, and *Verticillium dahlia* [30]. EIX (ethylene-inducing xylanase), identified in the nonpathogenic fungus *Trichoderma viride* originally, with β-1-4-endoglucanase activity, is an important class of elicitor in the GH11 family [31], and EIX-induced PTI immunity is not dependent on its enzymatic activity but the receptors LeEIX1 and LeEIX2 [32]. EIX homologues were isolated from plant pathogenic fungi, including *B. cinerea*, *F. graminearum* and *V. dahliae*, and FGSG_03624 from *F. graminearum*, BcXyn11A from *B. cinerea*, and VdEIX3, from *V. dahliae* also have the ability to trigger PTI immune responses and plant resistance to fungal infection [33,34,35,36]. In addition to GH11, other CWDEs-related GHs, including GH10, GH12, GH28, and GH45, have been proven to be fungal elicitors [37,38]. In addition to these CWDEs-related GHs, other fungal GHs have been proven to be fungal elicitors [39,40,41]. *B. cinerea* *BcCrh1*, a GH16 transglycosylase which catalyzes crosslinking of chitin and glucan polymers in fungal cell walls, has been proven to be an atypical fungal elicitor that functions in plant cytoplasm, and *Arabidopsis thaliana* expressing *BcCrh1* significantly increased resistance to *B. cinerea* [39].

Compared with GHs, CEs and PLs are relatively rarely reported to show elicitor activity [38]. SsCut1 and VdCUT11, two protein elicitors belonging to the CE family, were isolated from *Sclerotinia sclerotiorum* and *V. dahlia*, respectively [42,43]. SsCut1-treated plants showed induced expression of defense genes and significantly enhanced plant resistance to *S. sclerotiorum* and *Phytophthora sojae* [42]. Purified VdCUT11 induced cell death and triggered immune responses in *N. benthamiana*, cotton and tomato [43]. In addition, VdPEL1, belonging to the PL family, was identified in *V. dahlia* and purified VdPEL1 increased plant resistance to *B. cinerea* and *V. dahlia* [44].

#### 2.2.2. NEP1-like Proteins (NLPs)

Necrosis- and ethylene-inducing peptide 1 (Nep1)-like proteins (NLPs) with approximately 25k-Da, are firstly separated from culture filtrates of *F. oxysporum*, and are proven to be widely distributed in pathogenic fungi, such as *B. cinerea*, *B. elliptica*, *C. higginsianum*, *V. dahliae* and *Magnaporthe oryzae* [45,46]. Most NLPs contain the conserved peptide nlp20, which is therefore recognized by the PRR RLP23 and triggers the plant PTI immune responses [47,48]. Further research revealed that cytotoxic NLPs are able to bind to glycosyl inositol phosphoryl ceramide (GIPC) sphingolipids in plasma membranes of dicot plants, leading to tissue necrosis, then activating the defense response by promoting the accumulation of 1-aminocy clopropane-1-carboxylic acid synthase (ASC) and 1-aminocy clopropane-1-carboxylic acid oxidase (ACO) in plant cells [49].

#### 2.2.3. Harpin Proteins

Harpin proteins are heat stable, cysteine-free and glycine-rich proteins, and they can enhance plant resistance by eliciting a variety of immune responses such as HR, ROS bursts and ion fluxes [50]. Some harpin protein elicitors, such as Hrip1, MoHrip1 and MoHrip2, have been identified in *Alternaria tenuissima* and *M. oryzae* [51,52,53]. It has been demonstrated that Hrip1 from necrotrophic fungus *A. tenuissima* is able to induce cell death, pathogenesis-related (PR) genes expression, and (SAR) in tobacco [51]. Miao et al. showed that Hrip1 enhanced Arabidopsis resistance mainly by regulating the biosynthesis of defense related-jasmonic acid (JA) [54]. Both MoHrip1 and MoHrip2 from *M. oryzae* can induce cell death and defense responses, MoHrip1 regulated the levels of SA and GA in plants, and MoHrip2 induced the production of hydrogen peroxide and nitric oxide (NO) [52,53].

#### 2.2.4. Glycoprotein Elicitors

Glycoprotein elicitors including Elicitor1, Elicitor2, and Elicitor3 have been isolated from *Colletotrichum lagenarium*, and the chitinase activity of watermelon leaves can be increased upon the treatment of these elicitors [55]. Furthermore, glycoprotein elicitors can enhance resistance by inducing HR and lipid peroxidation. The treatment of specific glycoprotein elicitor GP66 from *M. oryzae* induced antioxidant activity and HR reaction [56]. Yang et al. purified a glycoprotein elicitor from *A. tenuissima*, and they found that it was able to enhance the resistance against tobacco mosaic virus in tobacco [57]. Glycoprotein elicitors can be divided into two types depending on the functional domain, one of which functions by sugar residue while the other utilizes amino acid residues [58], and the molecular mechanisms of glycoprotein elicitor-triggered immunity remain to be investigated.

#### 2.2.5. GPI-Anchored Proteins

Glycosylphosphatidylinositol (GPI) anchoring is one of the conserved post-translational modifications in eukaryotes, and GPI-anchored proteins are transported to the extracellular leaflet of the plasma membrane and cell wall [59,60]. Some studies have shown that GPI-anchored proteins from fungal plant pathogens, such as *Colletotrichum graminicola* and *M. oryzae*, are essential for fungal cell wall integrity and fungal pathogenicity [61,62]. A recent study showed that a Ser-Thr-rich GPI-anchored protein (SGP1) from *Ustilaginoidea virens*, the causal agent of rice false smut, is required for *U. virens* pathogenicity and is also a fungal PAMP triggering PTI immunity [63]. SGP1 is widely distributed among fungi and its homologues from many fungi can also trigger PTI immune responses [63]. Moreover, SGP1 treatment in *N. benthamiana* and rice significantly improved plant resistance to multiple fungal and bacterial pathogens [63], indicating good prospects of SGP1 elicitor in plant disease control.

#### 2.2.6. Secreted Proteins of Unknown Function

Some secreted proteins of unknown function from fungal plant pathogens are also proven to be elicitors triggering PTI immunity [64,65,66]. The fungal elicitor RcCDI1 identified in *Rhynchosporium commune*, whose homologues are found in a variety of fungi including *Zymoseptoria tritici*, *M. oryzae* and *Neurospora crassa*, can induce cell death in solanaceae [64]. Unlike the usual plant cell death, which is normally inhibited by the effector proteins AVR3a and PexRD2, cell death induced by RcCDI1 is not inhibited by these effector proteins, indicating RcCDI1-triggered immunity may be a novel immune pathway [64]. Furthermore, whether RcCDI1 induces other immune responses such as ROS and ethylene accumulation remains to be investigated. The *Valsa mali* elicitor VmE02, which has homologues in a lot of fungi, induces cell death, accumulation of ROS, callose deposition, activation of salicylic acid (SA) and JA-induced immune responses, and this process is dependent on some known components of the PTI signaling pathway, such as BAK1, SOBIR1, HSP90, and STG1 [65]. However, not all homologues of VmE02 can induce plant cell death, possibly due to the lack of a PRR recognition region or the diversity of protein sequences [65]. Further research revealed that the protein elicitor VmE02 is recognized by the PRR RE02 in *N. benthamiana*, and *RE02*-silenced plants show reduced resistance to fungal infection [67]. 

#### 2.2.7. Avirulence (AVR) Proteins

Unlike the above elicitors, AVR proteins usually interact with receptor proteins inside the plant cells and induce ETI responses [68]. The fungal AVR gene was first cloned in 1991 [69], and AVR proteins were identified in many plant pathogenic fungi, such as *Cladosporium fulvum*, *Puccinia graminis* f. sp. *tritici*, *Leptosphaeria maculans*, *M. oryzae*, *Blumeria graminis* f. sp. *hordei*, *Melampsora lini*, *Albugo candida*, and *Fusarium oxysporum* f. sp. *lycopersici* (Table 2) [70]. AVR proteins are recognized by resistance (R) proteins in plants and induce a series of immune responses such as intracellular ROS production and ion leakage, causing HR and SAR [70]. The identification of novel AVR proteins and their corresponding receptors (R proteins) is of great significance to plant disease control [70]. Two research studies published in *Science* in 2017 successfully cloned two important AVR genes (*AvrSr50* and *AvrSr35*) from *P. graminis* f. sp. *tritici*, the causal agents of wheat stem rust, and found that they activate wheat’s ETI immune responses by binding to wheat receptors (R proteins) Sr50 and Sr35, respectively [71,72], which provides significant insights into the control of wheat stem rust. 

**Table 1:** Summary of important fungal elicitors which trigger plant PTI immunity.

Type	Origin	Elicitor Name	Receptor	Receptor Type	Co-Receptor	Ref.
Saccharide	fungi cell wall	chitin/chitosan	OsCEBiP, LYK5	LysM-RLP, LysM-RLK	OsCERK1, CERK1	[73,74,75,76]
		β-Glucan	-	-	-	[27]
GH11	*Trichoderma viride*	TvEIX	LeEIX2	LRR-RLP	BAK1	[31,32,77]
	*Botrytis cinerea*	BcXyn11A	-	-	-	[34]
	*Fusarium graminearum*	FGSG_03624	-	-	-	[35]
	*Verticillium dahliae*	VdEIX3	NbEIX2	LRR-RLP	-	[36]
GH10	*Rhizoctonia solani*	RSAG8_07159, FGSG_11487	-	-	-	[78]
GH12	*B. cinerea*	BcXYG1	-	-	-	[79]
	*F. oysporum*	FoEG1	-	-	-	[80]
	*V. dahliae*	VdEG1	-	-	-	[81]
		VdEG3	-	-	-	
GH16	*B. cinerea*	BcCrh1	-	-	-	[39]
GH18	*Magnaporthe oryza*	MoChia1/MoChi	OsTPR1	Tetratricopeptide repeat protein	-	[40]
		MoChi/MoChia1	OsMBL1	Jacalin-related mannose-binding lectin	-	[41]
GH28	*B. cinerea*	BcPG1 to BcPG4, BcPG6	RLP42/RBPG1	LRR-RLP	-	[82]
GH45	*R. solani*	EG1	-	-	-	[83]
CE	*Sclerotinia sclerotiorum*	SsCut1	-	-	-	[42]
	*V. dahliae*	VdCUT11	-	-	-	[43]
PL	*V. dahliae*	VdPEL1	-	-	-	[44]
NLP	*B. cinerea*	BcNEP1, BcNEP2	RLP23	LRR-RLP	-	[84,85]
Harpin	*Alternaria tenuissima*	Hrip1	-	-	-	[51]
	*M. oryzae*	MoHrip1, MoHrip2	-	-	-	[52,53]
Glycoprotein	*M. oryzae*	GP66	-	-	-	[56]
GPI-anchored protein	*Ustilaginoidea virens*	SGP1	-	-	-	[63]
Secreted protein of unknown function	*Rhynchosporium commune*	RcCDI1	-	-	-	[64]
	*Valsa mali*	VmE02	RE02	LRR-RLP	-	[67]
	*F. graminearum*	Fg02685	-	-	-	[66]

**Table 2:** Summary of known fungal AVR proteins whose corresponding receptors have been identified.

AVR		Receptor			Ref.
Species	Name	Species	Name	Type	
*Albugo candida*	CCG28, CCG30, CCG33, CCG40, CCG67, CCG71, CCG79 and CCG104	Arabidopsis	WRR4A	TNL	[86]
	CCG45, CCG57, CCG61 and CCG70	Arabidopsis	WRR4B	TNL	
*Blumeria graminis* f. sp. *hordei*	AVRa1, AVRa6, AVRa7, AVRa9, AVRa10, AVRa13 and AVRa22	barley	MLA1, MLA6, MLA7, MLA9, MLA10, MLA13 and MLA22	CNL	[87,88,89]
*B. graminis* f. sp. *tritici* (*Bgt*), *B. graminis* f. sp. *Secalis*, *and B. graminis* f. sp. *Triticale*	AvrPm2, BgsE-5845 and BgtriticaleE-5845	wheat	Pm2	CNL	[90]
*Bgt*	AvrPm3A2/F2, AVRPM3B2/C2 and AVRPM3D3	wheat	PM3A, PM3F, PM3B, PM3C and PM3D	CNL	[91,92]
	AvrPm17	rye	Pm17	CNL	[93]
*Cladosporium fulvum*	apoplastic effectors, including Avr2, Avr4, Avr4E and Avr9	tomato	Cf-2, Cf-4, Hcr9-4E and Cf-9	PRR	[94,95,96,97,98]
*Fusarium oxysporum* f. sp. *lycopersici*	FoAvr2	tomato	I2	CNL	[99]
*F. oxysporum* f. sp. *melonis*	AvrFom2	melon	Fom-2	CNL	[100]
*Leptosphaeria maculans*	apoplastic AvrLm1	oilseed rape	LepR3	PRR	[101]
*Magnaporthe oryzae*	AvrPi9, Avr-Pi54, AvrPib, Avr-Pik, Avr-Pita and AvrPiz-t	rice	Pi9, Pi54, Pib, Pik, Pi-ta and Piz-t	CNL	[102,103,104,105,106,107]
	Avr-Pia and Avr1-CO39	rice	Pia-2/RGA5	CNL	[108]
	AvrPii	rice	Pi5-1, Pi5-2, Pii-1 and Pii-2	CNL	[109]
*Melampsora lini*	AvrL2-A, AvrM, AvrP and AvrP123	flax	L2, M, P and P2	TNL	[110,111]
	AvrL567 effectors	flax	L5, L6 and L7	TNL	[112]
*Puccinia graminis* f. sp. *tritici*	AvrSr27 and AvrSr35	wheat	Sr27 and Sr35	CNL	[72]
	AvrSr50	rye	Sr50	CNL	[71]
*P. polysora*	AvrRppC	maize	RppC	CNL	[113]
	AvrRppK	maize	RppK	CNL	[114]

## 3. The Receptors of Fungal Elicitors

### 3.1. Types of Receptors

The immune response induced by elicitors in plant species is dependent on the corresponding receptors on the cell membrane or within the cell. These receptors mainly contain the cell surface-localized pattern recognition receptors (PRRs) and intracellular nucleotide-binding leucine-rich repeat (NB-LRR) domain receptors (NLRs) [6,115]. 

#### 3.1.1. PRRs

As the key component of PTI signaling pathways, PRRs participate in the recognition of PAMPs, resulting in triggering PTI immune responses in plants [12]. PRRs are mainly divided into receptor-like protein kinases (RLKs) and receptor-like proteins (RLPs), and RLKs are comprised of a transmembrane helix, an extracellular domain, and a cytosolic kinase domain, while RLPs contain a short cytosolic tail instead of the cytosolic kinase domain [12]. The corresponding PRRs of some fungal elicitors have been identified mainly by virus-induced gene silencing (VIGS), microscale thermophoresis (MST) assay and co-immunoprecipitation (Co-IP), and these identified PRRs mainly belong to RLPs with leucine-rich repeat (LRR) ectodomains (LRR-RLPs) (Table 1). Interestingly, one chitinase elicitor MoChi/MoChia1 has been reported to induce PTI immune responses by interacting with two other membrane proteins, including tetratricopeptide repeat protein (TPR) and jacalin related mannose-binding lectin (MBL), instead of typical PRRs [40,41]. Unlike typical elicitors, MoChi/MoChia1 competed with OsMBL1 or OsTPR1 for chitin binding, thereby re-establishing the chitin-triggered immune response [40,41].

During PTI signaling pathways, PRRs usually require the involvement of co-receptors [116]. The somatic embryogenesis receptor kinase (SERK) family has been proven to act as co-receptors of PRRs, especially RLK-type PRRs [116]. BRI1-associated receptor kinase 1 (BAK1) is a well-known co-receptor of PRRs recognizing bacterial PAMPs, and it is also a co-receptor of LeEIX1 recognizing fungal EIX [77]. VIGS assay in *N. benthamiana* showed that BAK1 is also involved in PTI immune responses mediated by many other fungal elicitors [67,80,84,85], but whether BAK1 is a co-receptor of PRRs recognizing these fungal elicitors remains to be investigated. Chitin is the most studied fungal PAMP, and the rice chitin elicitor-binding protein (OsCEBiP) has been proven to be a significant chitin receptor in rice [75,76]. As OsCEBiP belongs to be RLPs and does not have intracellular domains, rice chitin elicitor receptor kinase 1 (OsCERK1), which is a lysin motif (LysM)-containing RLK, is proven to be a co-receptor of OsCEBiP for recognizing fungal chitin [18]. In Arabidopsis, the homolog of OsCEBiP, LYM2, was identified, but it did not induce immune responses [117]. Arabidopsis recognizes chitin to induce immune responses mainly lysin motif receptor kinase 5 (LYK5), and CERK1, which was able to phosphorylate LYK5 and triggered LYK5 internalization upon chitin treatment, is a co-receptor for LYK5 in Arabidopsis [73,74]. These results highlight the difference of PTI signaling pathways in different plants. 

In addition to being the receptors of pathogen PAMPs, PRRs can be recognized by apoplastic effectors to induce ETI immunity [118]. Tomato cf-9, which belongs to RLP-type PRR, was the first identified plant PRR in 1994 and recognizes the apoplastic effector Avr9 from *Cladosporium fulvum* [118]. Other plant PRRs, including Cf-4, Hcr9-4E and Cf-9 in tomato and LepR3 in oilseed rape, have been also proven to recognize fungal apoplastic effectors (AVR proteins) (Table 2).

#### 3.1.2. NLRs

NLRs, which evolved from a common ancestral prokaryotic adenosine triphosphatase, belong to the key and conserved components between plant intracellular innate immune systems and animal intracellular innate immune systems and mediate plant ETI immunity [119]. As the prominent intracellular immune receptors and R proteins, NLRs can be divided into three major classes, including the helical coiled-coil NLRs (CNLs), RPW8-L-like coiled-coil domain NLRs (RNLs) and Toll/interlecukin-1 receptor/resistance protein NLRs (TNLs) based on their variable N-terminal domains [119,120]. Based on the role of NLRs in plant ETI immunity, NLRs can also divided into sensor NLRs, which are involved in the recognition of intracellular effectors and helper NLRs, which do not regulate effectors but act as helpers or co-receptors to transduce immune signals from sensor NLRs [119,120]. RPS2 in Arabidopsis, which regulate effectors AvrRpm1 and AvrRpt2 from *Pseudomonas syringae*, and N in tobacco, which regulates effector p50 from tobacco mosaic virus (TMV), were the first identified NLRs in 1994 [118]. To date, numerous sensor NLRs recognizing fungal effectors have been identified in different plants and most of these NLRs belong to the CNL class (Table 2). Unlike PRRs, which mostly regulate specific PAMPs in a one-to-one way, NLRs recognize AVR proteins in one-to-one, many-to-one, or one-to-many ways [118]. The Arabidopsis WRR4A or WRR4B can regulate multiple effectors from *Albugo candida* [86], and wheat Pm2 can even regulate multiple effectors from different fungal pathogens [90]. In contrast, the effector AvrPii from *M. oryzae* can also be regulated by multiple rice NLRs [109]. Despite the great progress in the identification of NLRs that regulate fungal AVR proteins (Table 2), cloning of NLR genes remains challenging in some agricultural, horticultural and forestry crops, mainly due to their complex genomes and long growth cycles.

### 3.2. The Important Role of Immune Receptors Recognizing Fungal Elicitors in Disease Resistance Breeding

The breeding of disease-resistant cultivars is the most effective and economical method for plant disease control, and plant immune receptors directly or indirectly recognizing pathogen elicitors, mainly including PRRs and NLRs, have been proven to be key gene resources of disease-resistance breeding [121,122]. Traditional breeding or genetic engineering breeding can mediate the transfer of immune receptors from resistant plants to susceptible plants, thus conferring disease resistance in susceptible plants [122]. Transgenic overexpression of wheat NLRs, such as PM3A, PM3F, PM3B and PM3C, significantly increased wheat resistance to powdery mildew in the field [123]. In addition, transgenic expression of the barley MLA1, a NLR recognizing powdery mildew AVRa1, and tomato VE1, a RLP-type PRR recognizing apoplastic effector from *V. dahlia*, can induce Arabidopsis disease resistance [87,124], suggesting interfamily transfer of immune receptors mediated by genetic engineering breeding is a promising method for disease-resistance breeding. 

Plants are faced with the harm of many pathogens during growth and thus exploiting broad-spectrum disease resistance, which confers plant resistance against more than one pathogen species or against most races or strains of the same species, is a major goal of plant breeding [125]. Although most PRRs regulate specific PAMPs in the one-to-one way, a few plant PRRS have been proven to sense different elicitors from fungi, oomycetes or bacteria [118]. Transgenic overexpression of rice LYP4 and LYP6, which are dual functional PRRs sensing both bacterial peptidoglycan and fungal chitin, conferred rice resistance to both bacterial and fungal diseases [126], and the ectopic expression of Arabidopsis RLP23, a PRR recognizing different NLR proteins from fungi and oomycetes, in potato induced resistance to both fungal and oomycete diseases [48]. One NLR usually confers plant resistance to one or some pathogen strains which contain corresponding AVR proteins but pyramiding multiple NLRs can confer broad-spectrum disease resistance [125]. By introducing a transgene cassette of five resistance (NLR) genes into wheat, transgenic wheat showed broad-spectrum resistance to the notorious phytopathogenic fungus *P. graminis* f. sp. *tritici* [127]. Recently, transgenic rice carrying a designer rice NLR receptor RGA5^HMA2^ conferred resistance to *M. oryzae* carrying noncorresponding AVR proteins, indicating that introducing engineered NLR receptors can also be used in plant breeding for broad-spectrum disease resistance [128]. In addition to these receptors directly recognizing pathogen elicitors and functioning as positive regulatory factors of plant immunity, some receptor kinases, including BIR (BAK1-interacting receptor-like kinase) and FER (FERONIA), were proven to negatively regulate PRR signaling mainly limiting the formation of BAK1-receptor complex [118]. A recent study showed that genome editing mediated the inactivation of wheat receptor-like cytoplasmic kinase, TaPsIPK1, and conferred broad-spectrum resistance to stripe rust fungus *P. striiformis* f. sp. *tritici* without impacting important agronomic traits [129]. BAK1 is proven to be the co-receptor of some fungal elicitors or involved in PTI immunity-triggered by many fungal elicitors [67,77,80,84,85], and thus genome editing of these receptor kinases (BIR and FER) functioning as negative regulatory factors of plant PTI immunity may also be an important method for exploiting broad-spectrum disease resistance.

## 4. Signaling Pathways of Fungal Elicitor-Triggered Plant Immunity

**Figure 2:**
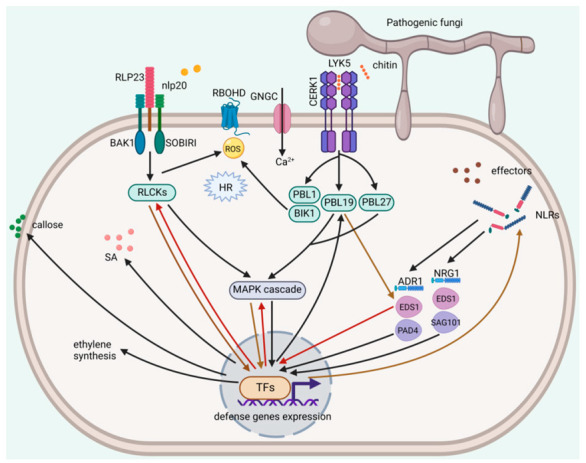
A model of the signaling pathways of fungal PAMPs-triggered PTI and fungal effector-triggered ETI. ROS, reactive oxygen species; HR, hypersensitive response; SA, salicylic acid. The figure is drawn by BioRender.

Upon the recognition between pathogen elicitors and plant receptors, these plant receptors, including PRRs and NLRs, will be activated, and then the signal cascades will be transduced in plant cells to trigger a series of PTI or ETI defense responses, which protect the plant from pathogen infection [130,131]. Noticeably, great progress has occurred in the signaling pathways of fungal PAMPs-triggered PTI and fungal effector-triggered ETI (Figure 2).

### 4.1. Fungal PAMPs-Triggered PTI

As the first line of innate immunity in plants, PTI immunity effectively controls the colonization of many pathogenic fungi [132]. The PRR complex plays a role in recognition with elicitors and signal transduction of PTI immunity [133], but receptors of most identified fungal elicitors remain unknown (Table 1). As one of the key structural components in the fungal cell wall, chitin is the most studied fungal PAMP, and great progress has occurred in the signaling pathways of chitin-triggered PTI (Figure 2). In *Arabidopsis*, the two LysM receptor-like kinases (LYKs), including LYK5 and CERK1, are thought to be the major chitin receptors, with LYK5 being phosphorylated by CERK1 and thus entering cells to induce downstream immune responses [73,74]. In addition, in vitro experiments have demonstrated that another LYK, LYK4, can interact with LYK5 to enhance the chitin-induced immune responses and improve plant resistance to fungi [134]. In contrast, two other LYKs, LYK2 and LYK3, are not to be involved in and negatively regulate the chitin-induced immune responses, respectively [135]. LIK1, an LRR-RLK, was also proven to be phosphorylated by CERK1 [136]. Unusually, the interaction between LIK1 and CERK1 inhibits CERK1 recognition of chitin and negatively regulates chitin-induced immune responses in *Arabidopsis* [136].

The receptor-like cytoplasmic kinases (RLCK), with no extracellular domain and no transmembrane helix, but only the cytosolic kinase domain, also has an important role in triggering PTI immune responses [137]. Typically, RLCKs are phosphorylated by PRRs, which in turn activate their immune functions that induce downstream immune signals [133]. In *Arabidopsis*, recognition of fungal chitin can activate a variety of RLCKs that induce PTI signals, including ROS burst, stomatal closure, callose deposition, MAPK cascades activation and defense genes expression. Ion flow is also one of the important signals of PTI, and GNGC has an important role in chitin-mediated calcium entry, and the study indicated a reduction in Ca^2+^ spikes in the GNGC mutant [138]. BOTRYTIS-INDUCED KINASE 1 (BIK1), an important RLCK, plays an important role in chitin-induced ROS production. The BIK1 phosphorylated by CERK1 has been reported to directly phosphorylate NADPH oxidase, the respiratory burst ortholog D (RBOHD), leading to ROS production [139]. Several phosphorylation sites have been identified on RBOHD to date, including the S347 site which can be phosphorylated in chitin-induced PTI [139]. RLCK VII-1, RLCK VII-4, RLCK VII-5 and RLCK VII-7 were also suggested to have the ability to induce ROS production [140]. ROS production induced by chitin was significantly higher in the WT than the *rlck vii-1*, *rlck vii-4*, *rlck vii-5* and *rlck vii-7* mutants [140]. RLCK VII-1 and RLCK VII-4 were specifically required for chitin-triggered PTI, whereas RLCK VII-5 and RLCK VII-7 are essential components of the immune pathways induced by a variety of PRRs [140]. ROS production is considered to be the starting signal of multiple immune pathways in plants, with the ability to enhance ion flow, NO production, and lead to the closure of plant stomata, which have an important role in the plant’s immune response [141,142,143]. Meanwhile, the strong scavenging system of plants ensures that ROS content in tissues will not be too high while ROS is produced at a high speed [144,145].

In addition to ROS production, activation of related RLCKs induces callose deposition. PBL1, a homolog of BIK1, was reported to be involved in chitin-induced callose synthesis, and *pbl1* mutant showed a significant reduction in callose deposition [146]. Another PBL protein, PBL27, a homolog of the cytoplasmic kinase OsRLCK185, was shown to be directly phosphorylated by CERK1 and had a role in inducing callose deposition [147]. Moreover, PBL27 acts as a MAPKKK kinase to phosphorylate MAPKKK5, which in turn activates MPK3/4/6, and several phosphorylation sites including S-617, S-622, S-658, S-660, T-677, and S-685 have been identified [148]. Phosphorylation of PBL27 by CERK1 enhances the activation of the MAPK cascade [148]. In MAPKKK5 mutants, chitin-induced callose deposition is significantly reduced, and therefore PBL27-induced callose deposition may be achieved through activation of MAPKKK5 [148]. Similar to PBL27, PBL19 activates MPK3/6 by phosphorylating MAPKKK5 [149]. The difference is that its phosphorylation sites are Ser-599, Ser-682, and Ser-692, whereas PBL19 phosphorylates the Ser-599 site [149]. Additionally, MPK6 activated by PBL19 is capable of enhancing immune signals by phosphorylating both the Ser-682 and Ser-692 of MAPKKK5 [149]. This is undoubtedly a positive feedback mechanism that has an important role in achieving strong disease resistance in Arabidopsis. It has also been reported that PBL19 can activate MPK4 by phosphorylating the MAPKKK MEKK1 [149]. Activation of the MAPK cascade normally phosphorylates downstream transcription factors and promotes the expression of defense genes. The transcription factor WRKY33 has been reported to act as a substrate for MPK3/6 and is phosphorylated to positively regulate the expression of *ACS2* and *ACS6* genes [150]. When infected by *B. cinerea*, ACS2 and ACS6 in *Arabidopsis* are involved in ethylene synthesis and plant disease resistance [150].

### 4.2. Fungal Effector-Triggered ETI

To overcome this first layer of defense (PTI immunity) and infect plants successfully, pathogenic fungi have evolved to secrete effectors into plant cells to inhibit plant PTI immunity, making plants more susceptible to pathogens, which is called effector-triggered susceptibility (ETS) [151]. Plants correspondingly produce resistance (R) proteins encoded by *R* genes, most of which are NLRs, to recognize some specific pathogen effectors (also called AVR proteins), thus triggering plant ETI immunity [152,153]. HR is a marked feature of plant ETI immunity and the activation of HR in infected sites markedly controls the colonization of pathogenic fungi [152,153]. Some plant R proteins can directly recognize fungal AVR proteins to induce ETI immune responses. For example, AvPi-ta from *M. oryzae* can be directly recognized by Pi-ta in rice, interacting with the LRR domain of Pi-ta and thus activating ETI immune responses in rice [154]. However, researchers found that most plant R proteins did not actually interact directly with AVR proteins. Thus, the defense hypothesis was put forward, which suggests that R proteins recognize AVR proteins through intermediate proteins and AVR proteins attack target proteins in plants and R proteins act as monitor proteins [155,156]. For example, the effector Avr2 from *C. fulvum* is not directly recognized by the R protein Cf-2 but is dependent on its target protein Rcr3 [155]. 

TNL-type NLRs meditated plant ETI immunity is usually dependent on members of the EDS1 family, including EDS1, PAD4 and SAG101, which share similar structural domains, a N-terminal α/β-hydrolase-like domain and a C-terminal EP domain containing the α-helical [157]. EDS1 was reported to function through its EP structural domain, in which a positively charged residue can be wrapped around its homologous protein to form a dimer [157,158]. In angiosperms, the helper NLRs ADR1 and NRG1 are generally thought to interact with EDS1-PAD4 and EDS1-SAG101, respectively, and mediate SA synthesis and cell death, respectively [159,160]. It was found that the Arabidopsis R protein, RPW8, which confers plant resistance to powdery mildew, induced SA- and EDS1-dependent defense responses, resulting in HR and the induced expression of defense genes [161]. This process was also dependent on PAD4, EDS5, and NPR1, but was inhibited by the MAPKK kinase EDR1, which controls plant exposure to spontaneous HR-like lesions (SHL) [161]. AVR proteins from *C. fulvum* can be recognized by Cf proteins in tomato, inducing ROS accumulation, HR, activation of ion channels and MAPKs, and expression of defense genes [162,163]. Cf-4 is an LRR-RLP that is dependent on the co-receptor BAKI to function. ETI induced by Cf-4 requires EDS1, but it is not clear whether helper NLR, SAG101 or PAD4 is involved in the ETI immunity [164]. The cytosolic heat shock protein 90 (Hsp90) is involved in plant ETI immunity-triggered by *C. fulvum* Avr2 by stimulating MAPKs, which in turn phosphorylates ACS and transcription factors to promote PR gene expression, ethylene synthesis, and HR production [165]. 

### 4.3. Convergent Pathways between Fungal Elicitor-Triggered PTI and ETI 

Arabidopsis receptor-like cytoplasmic kinase PBL19 has been proven to activate MPK4 by phosphorylating the MAPKKK MEKK1 during chitin-triggered PTI [149]. A recent study showed that PBL19 can also interact with EDS1, which is an important component of ETI upon treatment of fungal chitin, leading to the phosphorylation of EDS1 and strengthening the immune signal of plants [166]. This study suggests that EDS1 may be one of the key molecules linking PTI and ETI. In addition, Pruitt et al. also found that EDS1 and PAD4 in the ETI pathway were also involved in PTI trigged by fungal elicitor pg13, and reduction in ethylene production was observed in the *pad4* and *eds1* mutant compared to the wild-type response to pg13 elicitor [167]. Toll/interleukin-1 receptor (TIR) domains of NLRs were proven to be required for plant ETI immunity via their NADase activity [168,169], and a recent study showed that activation of TIR signalling is also involved in PTI immunity triggered by npl20 and other elicitors [170], which also highlights the noticeable PTI-ETI crosstalk. 

## 5. Conclusions and Prospects

This review summarizes recent advances in fungal elicitor-triggered plant immunity, including classification of fungal elicitors, the receptors of fungal elicitors, and signaling pathways of fungal elicitor-triggered plant immunity, which provides significant insights into plant–microbe interactions and plant disease control. Identification of novel elicitors is an important research topic in plant pathology because elicitors can be directly utilized in plant disease control and usually provide long-term plant protection [171]. Although many fungal elicitors have been identified (Table 1), little is known about elicitors in post-harvest fungal diseases mainly on fruit, which causes serious economic losses every year [172,173]. Similarities and differences between uncharted fungal elicitor-triggered plant immunity on fruit and the well-known fungal elicitor-triggered plant immunity on leaves remain to be investigated. In addition, only a few plant receptors that recognize fungal elicitors, especially PAMPs, have been identified so far, which undoubtedly poses a limitation to disease-resistance breeding. Thus, methods for identifying plant receptors need to be optimized, and the recent genome-wide silencing assay in the model plant *N. benthamiana* [174] may be a promising method of identifying receptors. 

Numerous studies have revealed the signaling pathways of PTI immunity triggered by fungal elicitors, especially the well-known fungal PAMP, chitin (Figure 2). PTI can be triggered both in the early and late stages of plant infection, whereas ETI triggering is concentrated in the late stages of infection and lasts for a longer period of time. However, the signaling pathways of fungal effector-triggered ETI have been relatively unexplored. The signaling pathways of ETI immunity triggered by bacterial effectors have been studied in detail [118,120], and whether these important components of the ETI signaling pathways triggered by bacterial effectors are involved in fungal effector-triggered ETI remains to be investigated. In addition, the crosstalk between PTI and ETI has been a hot topic in plant immunity research in recent years [118,175]. Thus, further revealing the molecular mechanisms of fungal PAMPs-triggered PTI and fungal effector-triggered ETI will uncover more convergent pathways between PTI and ETI. 

## Data Availability

Not applicable.
